# Barriers and incentives to orphan care in a time of AIDS and economic crisis: a cross-sectional survey of caregivers in rural Zimbabwe

**DOI:** 10.1186/1471-2458-6-27

**Published:** 2006-02-09

**Authors:** Brian H Howard, Carl V Phillips, Nelia Matinhure, Karen J Goodman, Sheryl A McCurdy, Cary A Johnson

**Affiliations:** 1 Center for Health Promotion and Prevention Research, University of Texas-Houston School of Public Health, Houston, USA; 2 Department of Public Health Sciences, University of Alberta, Edmonton, Canada; 3 Department of Sociology, University of Zimbabwe, Harare, Zimbabwe; 4 Africare Zimbabwe, Harare, Zimbabwe

## Abstract

**Background:**

Africa is in an orphan-care crisis. In Zimbabwe, where one-fourth of adults are HIV-positive and one-fifth of children are orphans, AIDS and economic decline are straining society's ability to care for orphans within their extended families. Lack of stable care is putting thousands of children at heightened risk of malnourishment, emotional underdevelopment, illiteracy, poverty, sexual exploitation, and HIV infection, endangering the future health of the society they are expected to sustain.

**Methods:**

To explore barriers and possible incentives to orphan care, a quantitative cross-sectional survey in rural eastern Zimbabwe asked 371 adults caring for children, including 212 caring for double orphans, about their well-being, needs, resources, and perceptions and experiences of orphan care.

**Results:**

Survey responses indicate that: 1) foster caregivers are disproportionately female, older, poor, and without a spouse; 2) 98% of non-foster caregivers are willing to foster orphans, many from outside their kinship network; 3) poverty is the primary barrier to fostering; 4) financial, physical, and emotional stress levels are high among current and potential fosterers; 5) financial need may be greatest in single-orphan AIDS-impoverished households; and 6) struggling families lack external support.

**Conclusion:**

Incentives for sustainable orphan care should focus on financial assistance, starting with free schooling, and development of community mechanisms to identify and support children in need, to evaluate and strengthen families' capacity to provide orphan care, and to initiate and support placement outside the family when necessary.

## Background

Sub-Saharan Africa is in an orphan-care crisis: 12.3 million children under age 15 have lost one parent (single orphans) or both parents (double orphans) to AIDS [1]. The burden of parental death from AIDS is greatest in southern Africa. In Zimbabwe, 19% of all children were orphans in 2003, four-fifths of them due to AIDS, leaving a population of 11.2 million to support 440,000 double and 820,000 single orphans [1-5]. With one-fourth of Zimbabwean adults infected with HIV [4,6] and antiretroviral therapies largely unavailable, AIDS will continue to reduce life expectancy – already down to 39 years from 63 years a decade ago – and increase orphan prevalence for years to come [2,7-11].

Zimbabwe's AIDS epidemic feeds and is fed by an economic meltdown marked by 70% unemployment, triple-digit inflation, a shattered agriculture sector, drastic cuts in social spending, and political uncertainty and paralysis [5-7,12-15]. In 2002, 49% of Zimbabweans were in need of emergency food aid, ranking worst in southern Africa [7,16]. The country's poverty rate has doubled since 1995 [17], and its 50 percent increase in under-5 mortality since 1990 is the largest in the world [18]. Isolated by Western donors critical of its government's human-rights record, Zimbabwe receives a tiny fraction of foreign aid to the region [18].

The dual disaster of AIDS and economic decline is straining the country's primary, preferred, most cost-effective, and previously well-defined and almost fail-safe system of orphan care – the extended family [2,5,7,19-23]. Within weakening patrilineal structures (including waning wife-inheritance customs), some fathers (of maternal orphans), aunts, and uncles are defaulting on traditional orphan-care responsibilities [5,24]. In increasing numbers, orphans are being fostered (used here in the sense of being taken in and raised, although Zimbabweans would not usually attach the term "fostering" to what they view as family arrangements) by relatives from the maternal side who are female, widowed, old, and poor, or by siblings – each a risk factor for deepening poverty [8,10,14,16,19,25-30].

Almost one-fourth of rural households are fostering orphans [2,21,31], including a growing percentage of the poorest households [3]. The numbers of street children and households headed by children, two largely unmonitored bellwethers of the crisis, are believed to be small but rising [2,5,14,19,22,24,32,33]. Alternatives are scant: The country's 45 registered orphanages house only 4,000 children [7], and while unrelated families may make mutually beneficial "voluntary fostering" arrangements (e.g. to facilitate a child's education or obtain domestic labor), crisis fostering in response to death or illness remains rare outside the kinship network [2,26,30,34].

While most orphans are still finding homes within their extended families, the costs of unassisted, state-of-emergency orphan care may be unsustainably high for children, caregivers, and society [8,14,16,23,29,35]. Starting with their ill parents' impoverishment, cumulative AIDS traumas leave orphans at high risk of malnutrition, emotional problems, illiteracy, economic and sexual exploitation, HIV infection, and future unemployment and poverty [10,36-41]. Rising school fees intensify the vicious cycle [40]: Primary-school enrollment rates have dropped precipitously, from 95% for boys and 90% for girls in 1999 to 67% and 63%, respectively, in 2003 [17,42]. Compared to other children, orphans are less likely to be in school, to stay in school during hard times, to be at the proper grade level, and to perform well [8,11,16,26,27,36,39,41]. Lack of stable, nurturing care exacerbates all of these risk factors, with potentially disastrous aggregate effects on society's public health, economic productivity, and social cohesion [1,3,27].

Many caregivers, especially the elderly, are impoverished, ill, tired, and emotionally drained from having cared for and buried relatives and taken in their orphans [22,43]. As the World Health Organization has noted, orphan care is "provided mostly in circumstances of diminished or non-existent forms of external support, be it familial or state-provided" [22,35]. Government programs are underfunded and difficult to access [7,29,35,44]; a study in 2000 found only 2% of households were benefiting from such public education, food, and health-care assistance, while family and community capacity was dwindling [45]. Without such support, a caregiver's illness or age-related frailty may thrust the foster child into the role of caregiver or head of household [33,46]. Moreover, given current population dynamics resulting from AIDS, the next generation of orphans will have far fewer grandparents as potential caregivers [31,47].

The limits of the extended family's sense of responsibility for orphans are largely unstudied. Evidence suggests that poverty and the prospect of having to pay school fees are barriers to fostering [40,48] and that mitigating factors, such as the presence of teenage siblings and nearby relatives to provide support, may make child-headed households more acceptable and likely [24]. Little is known about how factors such as finances, degree of relatedness, AIDS stigma, personal preferences, and gender and health of the child interact in orphan-care decisions [8,22,23,34,40,43,49,50]. We do not know whether extra-familial fostering remains infrequent because of community or personal preferences, a lack of effective mechanisms for prompting and supporting fostering, or simply a financial need to limit obligation. No published study from Zimbabwe has focused on caregivers' views of barriers to orphan care and incentives for overcoming them.

Answers to these questions can stimulate and inform policies and programs to avert what USAID calls "an impending calamity": that millions of children will grow up without the nutrition, education, and social nurturing necessary to sustain a healthy society [5]. This study explores the circumstances, needs, perceptions, and experiences of 371 caregivers in rural eastern Zimbabwe, including 212 who are fostering orphans, then briefly discusses implications for policies to strengthen the capacity of families and communities to care for their own.

## Methods

In August 2003, as part of a cross-sectional baseline survey, the aid organization Africare interviewed 371 primary caregivers of students at 34 primary and secondary schools in eastern Zimbabwe's rural Mutasa District. Two-thirds were selected at random from among participants in Africare's Community-Based Protection and Empowerment (COPE) for Children Affected by AIDS program, which planned (but had not yet begun) to provide school fees, psychosocial support, and income-generating assistance to households identified by school and community representatives as most severely affected by AIDS. The remaining third were caregivers of program participants' classmates, matched by grade level and gender. "Primary caregiver" was defined as the person the child would turn to first if s/he needed food, clothes, a personal item, affection, comfort, or guidance, and was identified by the child and confirmed by the caregiver in question.

A 114-item questionnaire included 62 closed-ended items exploring caregivers' demographic characteristics (including the presence of a spouse and of orphaned children), well-being, needs, resources, and perceptions and experiences of orphan care. (Fifty-two items not bearing directly on fostering were excluded from this analysis.) No formal ethics approval was required or sought for this program research. The questions were reviewed for content and appropriateness by a consultant from the University of Zimbabwe, discussed in depth with Africare managers and the data-collection staff (15 University of Zimbabwe social-science graduates), and pilot-tested with 50 caregivers, undergoing revision at each step. The endorsement and active support of local political, traditional, and school officials were sought and obtained. Written consent was obtained from respondents. No incentives for participation were offered. The questionnaire was administered in the local language, Shona, in settings intended to ensure privacy at the caregivers' homes. Of 402 caregivers selected for inclusion, 92.3% participated; two caregivers declined to participate, and data collectors were unable to reach the remaining 29. Data analysis using SPSS [51] describes the distributions of responses to questionnaire items in three respondent groups:

■ Group A ("foster caregivers"): 212 caregivers fostering double orphans.

■ Group B ("Africare parents"): 85 Africare project participants (from households severely affected by AIDS) who had not taken in orphans, though their own children were in most cases single orphans.

■ Group C ("control parents"): 74 parents from the rest of the school community, i.e. households that had not taken in orphans and were not selected for Africare assistance – perhaps the most promising pool of potential foster caregivers in this school-based study.

While groups B and C cannot be considered representative of any broader populations, their responses provide valuable descriptive context and insight into perceptions of fostering by parents within arm's reach of the orphan-care crisis. Where comparisons are of interest, confidence intervals for response-category proportions are reported at the 90% level.

## Results

### Who is fostering orphans? Who isn't?

Household size varied from one to nine children, with an average of four children in each of the three caregiver groups. Of the 148 caregivers whose prior relationship with their fostered child was determined, 53% were grandmothers (twice as often maternal as paternal), 22% aunts (about equally maternal and paternal), 14% siblings, 6% grandfathers, and 3% uncles. Among the children, there were no significant group differences in gender ratio, mean age, or grade level.

As shown in Table 1, most primary caregivers in all groups were women. More than one-third of fosterers (34%, CI (28%,39%)) were ages 60 or older, vs. 4% (CI (2%,6%)) of non-foster caregivers, and 15% were in their 70s or 80s. Ten foster caregivers (5%) were in their teens; six of them (two brothers, two sisters, and two aunts of the fostered children) were 17 or younger.

Many foster caregivers did not have a spouse, an important risk factor for poverty: 56% (CI (50%,62%)) were married, compared to only 31% (CI (22%,40%)) of Africare parents and 78% (CI (69%,86%)) of controls. More than one-third of foster caregivers were widowed.

About four-fifths of caregivers in each group had at least a primary-school education. A majority of foster households depended on subsistence farming for their livelihood. One-fifth reported no income-producing occupation, compared to 12% of controls. Foster households were about half as likely as control households to be headed by skilled or general wage employees, 20% (CI (15%,25%)) vs. 38% (CI (28%,48%)). Among Africare parents, only 11% (CI (7%,19%)) had such a steady source of income.

### Financial, physical, and emotional well-being

Most families in all groups were struggling to feed themselves. Those fostering orphans were somewhat worse off economically than control households. The direst problems were reported by Africare parents; these households severely affected by AIDS were least likely to contain a wage earner, to get enough to eat, to eat meat, to own cattle and household assets, and to live in brick housing.

In each group, more than one-third reported experiencing hunger in the household twice or more a week. Only 14% (CI (10%,18%)) of foster caregivers said their families always got enough to eat, compared to 6% (CI (2%,12%)) of Africare parents and 26% (CI (23%,42%)) of controls. Less than half of all caregivers said they ate three meals per day. Less than 10% in any group reported eating meat more than once a week, and more than one-third said they never consumed meat. In this region, ability to afford meat is a good proxy for the likelihood that the household can obtain adequate nourishment.

When asked to whom they could turn for help, 59% of all caregivers said "no one." Most others cited relatives or neighbors; only 2% mentioned the government, local leaders, or nongovernmental organizations.

In each group, 19% of households contained at least one child ages 6–17 who was not in school, most often (52%) because of inability to pay school fees. These children made up 8% of the school-age population in surveyed households. Of these out-of-school children, 14% were ages 7–12 and 26% were ages 13–15, ages at which even late starters and early finishers should be in school. Of the 12 out-of-school children ages 7–12, 10 (83%) lived in households fostering orphans.

**Figure 1:**
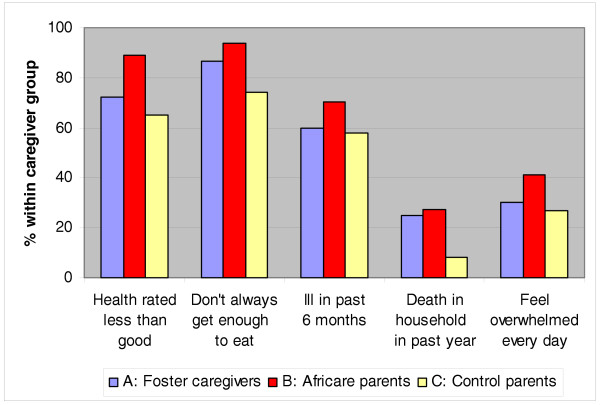
**Caregiver well-being, by group (n = 371)**. Financial, emotional, and health stresses were higher in households fostering double orphans (A) than in households without orphans (C). In households severely affected by AIDS (B), most of which contained single orphans, caregivers reported the most severe problems.

Like financial measures, proxies for physical and emotional well-being showed foster caregivers struggling at a level between the Africare parents and the somewhat better-off controls (Figure 1). Only 28% (CI (23%,33%)) of foster caregivers described their health as good (vs. 11% (CI (6%,19%)) of Africare parents and 35% (CI (26%,45%)) of controls), while 22% (CI (18%,28%)) of foster caregivers rated their health as poor or very poor. Two-thirds of foster caregivers had been ill during the previous six months, and almost half reported a current illness in the household. About 17% had been ill for at least three months during the previous year. One-fourth (CI (20%,30%)) reported a death in the household within the previous year (compared to 27% (CI (19%,36%)) of Africare parents and 8% (CI (4%,15%)) of controls). Daughters, sons, and daughters-in-law of the primary caregiver made up almost half of the deaths in fostering households.

Among foster caregivers, 29% reported feeling overwhelmed every day by responsibilities (41% of Africare parents, 27% of controls). More than half of all caregivers expected life for their children and grandchildren to be worse than their own. This form of pessimism was lowest among foster caregivers (38%) and highest among Africare parents (54%). Despite this evidence of emotional stress, when caregivers were asked what would most help them, they mentioned almost exclusively financial assistance, topped by school fees (84%), food (70%), and help with income generation (49%). All 65 caregivers who identified themselves as chronically ill ranked access to education as one of their greatest concerns about their children's future without them.

### Attitudes toward fostering orphans

Table 2 shows caregivers' responses to questions about their willingness to foster, important factors in a decision to foster, reasons not to foster, and experiences and perceptions of fostering.

#### Willingness to foster

All but eight (2%) of 357 caregivers responding said they were willing to foster a child, regardless of whether they were already fostering. Willingness to foster was highest for grandchildren and declined with increased distance in relatedness. The sharpest drop-off occurred at the family line: While three-fourths of caregivers in all groups would foster a relative's child, fewer than half would take in a friend's child. Still, about one-fourth of caregivers said they would foster even a stranger's child.

#### Important factors in a decision to foster

Foster caregivers said the decision to foster was made most often by the woman of the house (38%), frequently the only adult in the household. In 25% of cases, it was the extended family's decision. About 8% of foster caregivers said the decision to foster provoked strong disagreements within the family. The main reason foster caregivers gave for fostering was that there was no one else to care for the orphan (71%), and 11% cited family duty. Love of the child was the main reason for 16%.

Among important factors in deciding whether to foster a child, all three groups ranked degree of relatedness and financial capacity at the top. Among non-fosterers, financial concerns were cited more often, 55% (CI (48%,62%)) to 48% (CI (41%,55%)). Few caregivers said the child's age and gender were important considerations; among those with an age preference, children younger than 2 were least desired.

Asked to select the single most important factor in a fostering decision, caregivers again cited financial capacity and degree of relatedness most frequently. Considering the frequent assertion that community or personal preference limits fostering to blood relatives, it is worth noting that more than two-thirds of caregivers said other factors were more important.

#### Reasons not to foster

Lack of money was the only frequently cited barrier (43%) to fostering orphans whom respondents viewed as their responsibility. About half of all caregivers said nothing could prevent them from fostering such a child.

Responses in all groups reflected high levels of AIDS-related anxiety and stigma as well as compassion. One-third of all caregivers (47% of controls) said they were "very worried" about becoming infected with HIV. More than 90% of all caregivers were willing to care for a relative with AIDS in their homes, but almost half said they would want to keep it a secret if a family member had AIDS. More than one-fourth of all caregivers said an HIV-positive teacher should not be allowed to continue teaching, even if s/he were not ill, and 40% said they would not allow their children to play with an HIV-positive child. Caregivers with less education were more likely to hold these views than better-educated caregivers.

#### Fostering experience and perceptions

Two-thirds of foster caregivers said they experienced financial difficulties as a result of fostering. Three-fourths said their fostered child brought no resources into the household. Asked what the best thing about fostering was, 53% said it was the satisfaction of doing their duty, followed by the satisfaction of helping the child and the joy of having the child in the family.

Perceptions of fostering in general were mixed. The statement "Many orphans who are taken into new families are not treated well and would be better off on their own or in orphanages" found majority agreement in all groups – 60% overall, 81% among married controls whose families always got enough to eat. Given the limitations of quantitative surveys and of this question, it is unclear whether this points to unreported maltreatment, a general perception of fostering as less than ideal, or rationalizing by non-fosterers. Only about one-fifth of caregivers (one-fourth of controls) agreed that "When a parent has to make choices about school and food, biological children should be considered first" (before fostered children).

Most caregivers said those who take in orphans gain respect in the community; parents in the control group were least likely to hold this belief, 77% (CI (67%,85%)) vs. 83% (CI (79%,86%)) of the other groups. Most caregivers expressed optimism about an orphan's life chances. Control parents were by far the most pessimistic: 29% (CI (20%,39%)) disagreed with the statement that "If taken into good homes, orphans will probably live good, successful lives," vs. 14% (CI (10%,19%)) of other caregivers. Among married controls whose families never go hungry, 38% expressed pessimism about orphans.

### Beyond the family: Control parents and extra-familial fostering

Compared to other caregivers, Group C controls were most likely to have a spouse and a wage-earner in the household (Table 1) and to be in good health. This makes them the most likely source of viable, as-yet untapped foster caregivers. It should not suggest that they are an uncaring or AIDS-phobic elite: Many experienced hunger regularly; 24% were willing to take in any child; and they were the group most likely to be willing to care for a relative with AIDS and to let their children play with HIV-positive friends.

On the other hand, as shown in Table 2, control parents were most likely to be concerned about the orphan's health (including HIV status), age, and gender. They ranked relatedness as the most important factor in a fostering decision, 34% (CI (25%,44%)) to 25% (CI (17%,34%)) over finances. They were most likely to want to keep a relative's illness with AIDS a secret, to consider the possibility that the child might have HIV a good reason not to foster, and to be "very worried" about becoming infected with HIV.

**Figure 2:**
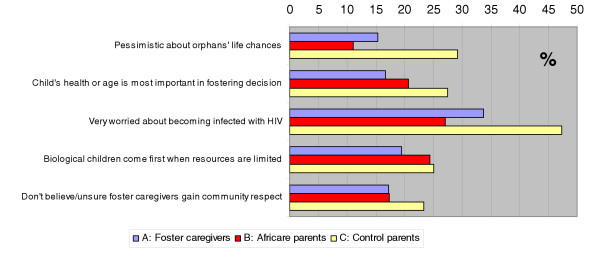
**Caregiver perceptions that might inhibit fostering, by group (n = 363)**. Parents who were not caring for orphans (C), perhaps the most likely source of untapped foster caregivers, were more pessimistic about orphans' life chances, more likely to believe biological children should be given preference, and least likely to believe that caregivers who take in orphans gain community respect. Perceptions of this kind may limit potential caregivers' motivation to step forward for fostering, especially extra-familial fostering.

Their mixed views related to fostering are illustrated in Figure 2. They were more pessimistic about orphans' life chances, more likely to believe biological children should be given preference, and least likely to believe that caregivers who take in orphans gain community respect. Perceptions of this kind may be important factors in limiting potential caregivers' motivation to step forward for fostering, especially extra-familial fostering.

## Discussion and conclusions

Caregivers' overwhelming willingness to foster orphans, motivated by family obligation and compassion and constrained primarily by poverty, is a strong foundation for a system of orphan support that could ensure a home for every child. Their responses suggest that the greatest barriers to fostering are problems of money and organization and that policies to encourage good orphan care should focus on financial assistance and the development of local mechanisms for supporting and supplementing families, the country's greatest strength.

The study confirms the literature's descriptions of foster caregivers as disproportionately female, older, and single, and as struggling financially, physically, and emotionally [27]. To raise healthy children, most need financial assistance. A fostering stipend, supplemented as needed by assistance with food, health care, and income generation [52], would be an enormous administrative and funding challenge to undertake on a national scale, but it could begin with external assistance for community-based efforts [53,54].

The most urgently requested form of financial assistance is educational subsidies. Free schooling would encourage orphan care by more nearly balancing the costs and benefits of fostering as perceived by caregivers [34]. It would also help to ensure that care arrangements are sustainable, reducing the risk of repeated migrations [46]. A publicly financed school system guarantees access to all; until such a system is implemented, school fees, which create a barrier to fostering and a trap of lifetime poverty for poor children, should be subsidized for all children in need. Even after recent enormous increases that are prohibitively high for many families, school fees of about $18–$40 U.S. per child per year [55,56] for secondary school (less for primary) mean that a child's prospects in life can be dramatically improved for an easily administered investment of $200.

Some of the most vulnerable children in this study are single orphans living with the surviving parent in households in the process of AIDS impoverishment. Their economic circumstances may actually improve somewhat after they become fostered double orphans. Support for these children must not wait until then.

As the range of need in all three groups suggests, orphan status by itself is not necessarily the most important marker of child vulnerability. It is widely understood that targeting assistance only to children orphaned by AIDS is unfair, produces resentment, stigmatizes the children, and should be avoided [9]. Whether aid should be targeted to orphans (from all causes) or to all children who are vulnerable (in specified ways) may depend on the intent of the policy or program and the kind of help it provides. The World Bank argues that for most kinds of economic assistance, poverty is a better targeting criterion than orphanhood [14]. For example, given Zimbabwe's history of once-high rates of school enrollment even among the poor, lower enrollment rates among orphans were "likely related to problems specific to being an orphan," such as discrimination and life disruption, rather than absolute financial need. Subsidizing school fees, textbooks, and uniforms for orphans might waste funds on orphans who don't need such financial assistance (though they might need other kinds of support to stay in school) and might encourage "opportunistic redistribution" of orphans so other household members can take advantage of their financial benefits, the World Bank contends. Considering the sheer need, this seems a minor price to pay, and in light of Zimbabwe's widespread impoverishment and dropping enrollment rates, the premise may no longer be valid. But the general point – material assistance to the neediest will help the neediest orphans as well – is valid and would apply to any benefit that poor non-orphans also need, such as school fees, health care, and supplemental food. It would not apply to orphan-specific benefits, such as grief counseling.

Still, orphan status could be a useful screening point for financial need as well as "problems specific to being an orphan," and if, as here, the policy or program intent is to encourage the fostering and healthy development of orphans (rather than to increase general school enrollment), then linking educational subsidies with orphan programs may make sense. Local pilot programs should test and determine how to prioritize double orphans, single orphans, and other vulnerable children.

This study found no reservoir of economically secure households that must simply be persuaded to take in orphans. Based on the control group's reservations about fostering, two-parent households that are not fostering orphans may be a good target for educational outreach emphasizing AIDS-stigma reduction and the rewards of fostering, in addition to financial support starting with guaranteed school fees.

Kinship obligation is clearly the most important motivation to foster, but its absence is not an insurmountable barrier in cases where no viable extended family exists. Most caregivers said other factors – most often financial capacity – mattered more than relatedness. The factors that lead to voluntary fostering arrangements with non-relatives – mutual need and mutual benefit – may be instructive for crisis fostering: At the least, encouraging extra-familial fostering will require enough financial assistance to suggest that an outsider's child is not an irresponsible investment of a household's scarce resources [34].

The extended family is the only effective structure in place to prompt and support decisions on orphan care. NGOs should consider providing technical expertise and long-term funding to encourage the formation and evaluation of locally accountable community mechanisms to routinely respond to families in need, to identify and intervene early in support of children in households severely affected by AIDS, to evaluate and strengthen families' capacity to provide good orphan care, and to initiate placement outside the family when necessary. These mechanisms should serve all orphans, regardless of cause, and should reinforce related efforts, such as identification of vulnerable children (e.g. through follow-up by HIV testing and palliative-care services) and existing education and social services. Above all, such mechanisms must avoid damaging the extended family's sense of responsibility for and control over decisions regarding the care of their young.

On a backbone of economic and organizational capacity-building, programs can better address other issues raised by the caregivers' responses. One such issue is the persistence of fear and stigma surrounding AIDS. While caregivers did not cite them as major factors in a fostering decision, they may be secondary barriers, e.g. by inhibiting initiative to foster an "AIDS orphan," especially from outside the family. Wariness of people with AIDS was inversely associated with educational level, suggesting that underlying attitudes might be affected through educational outreach, e.g. using HIV-positive peer educators.

The most urgent research need is for locally based pilot programs that test effective, harm-free levels and mechanisms of financial and organizational support in areas where child-headed households are occurring. A second priority is to monitor the prevalence of child-headed households, street children, and children denied access to school by fees.

Finally, affordable and accessible antiretroviral medications will be a huge step toward ending the orphan-care crisis. In orphan care, as in fighting the underlying disease, nothing beats prevention.

This convenience sample lacks the power to disentangle the many ways in which AIDS, poverty, and community and personal preferences interact with regard to the care of orphans. A study of practices, rather than attitudes, among those who foster orphans and those who do not would provide better insights into how to increase the uptake of orphans within and outside the kinship network. Many questions about how orphan-care decisions arise and are confronted could be explored in greater depth by using qualitative methods. In excluding households that are too poor, sick, or disarrayed to send their children to school, this study almost certainly understates the severity of the crisis, while excluding households without children neglects a potential part of a solution. In addition, study methods may have influenced some survey responses, e.g. by encouraging responses focusing on sources and types of assistance that respondents consider relevant to or available from the aid organization funding the research. These limitations point toward opportunities for useful program research.

Among non-fostering control parents, the current study is not able to distinguish between those who had refused to foster an orphan, those who had not acted on an opportunity to volunteer to foster an orphan, and those who had never faced a fostering decision. This distinction would be useful in interpreting and making programmatic use of their responses. It seems likely that in most cases, AIDS had not yet struck close enough to home to make fostering a family duty, no external mechanism had confronted them with the choice of fostering from outside the family, and no financial assistance had made doing so seem feasible. Regardless of their previous experiences, however, their views of what is important in a fostering decision can help inform efforts to facilitate a future decision and attempt to foster.

While Zimbabwe's unique combination of economic crisis, AIDS dispersal, and political isolation will limit generalizability of these findings, identified themes may contribute to building an international consensus on responsibilities and strategies for orphan care anywhere. In decisive ways for Zimbabwe, orphans are the foreseeable future and strengthened families their best hope. How communities, the country, and the world move to help the too-old and too-poor nurture the too-young through the double disaster of AIDS and poverty will shape the nation's health and prospects for generations to come.

## Competing interests

The author(s) declare that they have no competing interests.

## Authors' contributions

CAJ conceived the study and oversaw its design. BHH designed the study, organized and led data collection, analyzed and interpreted the data, and drafted the manuscript. NM assisted in study design, supervised data entry, and critically reviewed the manuscript. CVP, KJG, and SAM helped analyze and interpret the data and revise the manuscript. All authors read and approved the final manuscript.

## Pre-publication history

The pre-publication history for this paper can be accessed here:



## Figures and Tables

**Table 1:** Caregiver characteristics (n = 371)

	**Group A: Foster caregivers ** **(n = 212) (%)**	**Group B: Africare parents** ** (n = 85) (%)**	**Group C: Control parents** ** (n = 74) (%)**
**Gender**			
Female	85	94	89
Male	15	6	11

**Age**			
< 20 years	5	0	0
20–29 years	14	5	5
30–39 years	12	29	30
40–49 years	17	49	42
50–59 years	19	13	18
60+ years	34	5	5

**Marital status**			
Married	56	31	78
Divorced	6	4	1
Never married	5	1	0
Widowed	37	65	20

**Education (level completed)**			
< Primary school	22	17	13
Primary school	56	71	71
Secondary school	19	12	17
College/university	3	1	0

**Main occupation of head of household**			
Subsistence farmer	54	58	47
Informal trader	4	8	1
Skilled worker	7	2	11
General worker	13	9	27
Business	0	0	1
Part-time jobs	1	1	0
Traditional healer	1	0	0
None	20	21	12

Note: May not sum to 100% because of multiple responses and rounding.

**Table 2:** Attitudes related to fostering, by caregiver group (n = 371)

	**A: Foster caregivers (%)**	**B: Africare parents (%)**	**C: Control parents (%)**
Willing to foster:*			
Grandchild	92	84	87
Sister or brother's child	84	82	89
Other relative's child	77	73	80
Friend's child	47	47	46
Neighbor's child	34	36	44
Child from community	31	35	33
Child from church	30	31	30
Child from home region	28	29	30
Stranger's child	26	23	24
Any child in need	10	3	1
Would not foster a child	1	5	1

**Important factors in a fostering decision:**			
How closely the child is related to you	53	49	44
Financial resources or assistance	46	55	55
Child's health	21	18	26
How well you know the child	16	11	15
Child's behavior	15	20	12
Child's age	14	13	18
Child's intelligence	10	11	11
Child's gender	4	4	4

**Single most important factor:**			
How closely the child is related to you	34	26	34
Financial resources and assistance	32	35	25
Child's health	9	12	15
Child's age	7	9	12
Child's gender	7	2	4
Child's plight	2	2	3
All are important	4	7	4

**What could prevent fostering suitable child:**			
Nothing could prevent it	54	45	43
Lack of financial resources	40	46	47
If child is HIV-positive	7	10	10
If child is ill	5	8	3
If child is disabled	3	8	4
Sacrifices biol. children would have to make	3	3	3

**Good reasons, other than financial, not to foster:**			
Will cause problems in household	29	28	18
Not my responsibility	18	16	19
Don't have strength	17	16	15
Child might have HIV/AIDS	13	7	16

**"If taken into good homes, orphans will probably live good, successful lives."**			
Agree	80	85	68
Disagree	15	11	29

**Main reason for taking in an orphan:**			
There was no one else to care for the child	71	NA	NA
Love of the child	16		
Family duty	11		

**Difficulties resulting from fostering:**			
Financial difficulties	64		
Emotional strain or ill health	4	NA	NA
No difficulties	30		

**Best thing about fostering:**			
Satisfaction of doing your duty	53		
Satisfaction of helping the child	37		
Joy of having this child in the family	22	NA	NA
Material benefits	11		
Closer relationship with child	10		
Respect of family and community	6		
A full life	4		

Note: May not sum to 100% because of multiple responses and rounding.

* Caregivers who said "Any child in need" were also counted in other categories.
